# Health facility preparedness for cholera outbreak response in four cholera-prone districts in Cameroon: a cross sectional study

**DOI:** 10.1186/s12913-019-4315-7

**Published:** 2019-07-08

**Authors:** Jerome Ateudjieu, Martin Ndinakie Yakum, Andre Pascal Goura, Sonia Sonkeng Nafack, Anthony Njimbia Chebe, Joliette Nguefack Azakoh, Benjamin Azike Chukuwchindun, Eugene Joel Bayiha, Corine Kangmo, Gnodjom Victorin Boris Tachegno, Anne-Cécile Zoung Kanyi Bissek

**Affiliations:** 1 M.A. SANTE (Meilleur accès aux soins de Santé), P.O. Box 33490, Yaoundé, Cameroon; 2 Department of Biomedical Sciences, University of Dschang, P.O. Box 067, Dschang, Cameroon; 3 Division of Health Operations Research, Ministry of Public Health, Dschang, Cameroon

**Keywords:** Cholera, Preparedness, Hygiene, Sanitation, Water, Surveillance, Health facility, WaSH

## Abstract

**Background:**

The risk of cholera outbreak remains high in Cameroon. This is because of the persistent cholera outbreaks in neighboring countries coupled with the poor hygiene and sanitation conditions in Cameroon. The objective of this study was to assess the readiness of health facilities to respond to cholera outbreak in four cholera-prone districts in Cameroon.

**Methodology:**

A cross-sectional study was conducted targeting all health facilities in four health districts, labeled as cholera hotspots in Cameroon in August 2016. Data collection was done by interview with a questionnaire and by observation regarding the availability of resources and materials for surveillance and case management, access to water, hygiene, and sanitation. Data analysis was descriptive with STATA 11.

**Principal findings:**

A total of 134 health facilities were evaluated, most of which (108/134[81%]) were urban facilities. The preparedness regarding surveillance was limited with 13 (50%) health facilities in the Far North and 22(20%) in the Littoral having cholera case definition guide. ORS for Case management was present in 8(31%) health facilities in the Far North and in 94(87%) facilities in the littoral. Less than half of the health facilities had a hand washing protocol and 7(5.1%) did not have any source of drinking water or relied on unimproved sources like lake. A total of 4(3.0%) health facilities, all in the Far North region, did not have a toilet.

**Conclusions:**

The level of preparedness of health facilities in Cameroon for cholera outbreak response presents a lot of weaknesses. These are present in terms of lack of basic surveillance and case management materials and resources, low access to WaSH. If not addressed now, these facilities might not be able to play their role in case there is an outbreak and might even turn to be transmission milieus.

**Electronic supplementary material:**

The online version of this article (10.1186/s12913-019-4315-7) contains supplementary material, which is available to authorized users.

## Introduction

Cholera remains a major public health problem in Cameroon with several episodes of outbreak registered since 1971 [1, 2]. According to the national surveillance data (unpublished sources), the most recent cholera outbreaks occurred in 2014, 2015 and 2018. Between 2000 and 2012, a total of 43474 cases of cholera were reported in Cameroon with 1748 deaths giving a case fatality rate of approximately 4.0% [3]. Based on literature, the most affected rural districts were in the Lake Chad basin in the Far North region while the most affected urban districts were in Douala, Littoral region [3–6].

The risk of cholera outbreak in Cameroon remains high. This is due to the massive population exchange with, and persistent cholera outbreaks in the neighboring countries like Nigeria, Central Africa Republic, and Chad coupled with limited access to water, sanitation, and hygiene (WaSH) in the country [4, 7, 8]. According to the results of Multiple Indicators Cluster Survey 5(MICS5) conducted in 2014, 65.1% of the population did not have access to improved unshared latrines in Cameroon [9]. To the best of our search, no data exist in Cameroon on the situation of WaSH at Health facility.

An effective cholera control incorporates three major phases related to preparedness, response, and recovering (post-epidemic) phases [10]. Although each of these phases is equally very important, the level of preparedness is the backbone of cholera control since the success of cholera outbreak response depends largely on it. Based on existing evidence, the highest cholera case fatality in Cameroon is always registered at the beginning of each outbreak [3]. This is probably uncovering weaknesses of preparedness. Preparedness is a multi-sectoral, multi-disciplinary, and is implemented at all levels of the health system (community and health facilities) [10]. Also, preparatory interventions are principally focused on reinforcing surveillance, training, prepositioning of supplies for case management, and improving WaSH [10]. According to the Cameroon national cholera contingency plan, main strategies for cholera outbreak response include: surveillance, case management, training, communication for development, improving access to WaSH, vaccination, coordination, operational research, resource mobilization and monitoring. However, the monitoring and evaluation of these interventions are not done and information on the preparedness is usually lacking.

During cholera epidemics, patients are rushed to the health facilities for treatment. The surveillance system in place must be able to detect the outbreak on time and the health facility on its part must have sufficient resources to manage cases to quickly stop the spread of the disease and reduce the death rate. On the other hand, if the hygiene and sanitation conditions in the health facilities are not good, it can lead to the spreading of the disease to other patients and health staffs [11, 12]. As a consequence, this may increase the attack and death rates of the outbreak. A study in the Far North region of Cameroon revealed that a good number of deaths from cholera occurred in the healthcare facilities [13]. The objective of this study was to evaluate the level of preparedness of health facilities to cope with any sudden cholera outbreak in four cholera hotspot districts in Cameroon. It aims to call the attention of health authorities on important elements that may be consider to reduce the death rate of cholera during outbreaks.

## Methods

### Study design

This was a cross sectional descriptive study targeting all health facilities in Kousseri, Mada, Deido and Nylon health districts of Cameroon. Data reported in the manuscript was collected in the framework of needs assessment that was used to improve the preparedness of health facilities in Cameroon by health authorities. Data was collected during August 2016 with the help of questionnaire and by observation on cholera surveillance, cholera case management, and access to WaSH. Data analysis was purely descriptive and done with STATA 11.

### Study settings

The study was conducted in Kousseri, Mada, Deido and Nylon health districts of Cameroon. These were selected because they were the most affected urban (Deido and Nylon) and rural (Kousseri and Mada) health districts during the ten previous cholera outbreaks in Cameroon. Deido and Nylon health districts are found in the economic headquarter of Cameroon, Douala, where access to some basic necessities such as water, road network, electricity, healthcare (geographical access), and schools is quite improved compared to Kousseri and Mada. Also, Kousseri and Mada are characterized by bad roads, limited access to electricity, long distance from home to the healthcare centers and poor access to good water sources. Furthermore, Kousseri and Mada are partly affected by the Bokoh Haram terrorism and movement slightly restricted to some sites and during certain period of the day. Some health facilities in Kousseri and Mada Health districts received patients from neighboring countries like Chad and Nigeria. This makes them most vulnerable to cholera case importation. Data was collected in 2016 when there was no outbreak in Cameroon so as to better appreciate the preparedness.

### Type of health facilities in Cameroon

Health facilities in Cameroon are either governmental (public) or private and are classified into five [5] categories. Starting from the based, we have the *Category 5* which is an integrated health centers (IHC) headed by a senior nurse and Sub-divisional hospitals headed by either a nurse or a medical doctor. The different between IHC and CMA is that CMA has a physician among the staff but the medical services are essentially the same which are the Minimum Package of Health care Activities (MPA). *Category 4* is represented by the District Hospital which is the first reference hospital headed by a medical director. It offers the complimentary package of activities. *Category 3* is represented by Regional hospital which is the second level of healthcare reference in charge of specialized health services at the level of the region. *Category 2* is represented by Central hospital in which the technical platform is higher than that of regional hospital and forms the third level of health care reference in Cameroon. *Category 1* is General hospital that included university teaching hospitals and more specialized services. This is the last level of health care reference system in Cameroon and handles cases that the central hospital cannot handle with more specialized equipment and knowhow.

### Sampling

In each selected district, all healthcare facilities legally registered were included into the study.

### Data collection

The cholera preparedness checklist was developed from review of international guidelines for cholera preparedness and previous publications on the topic [14–18]. However, to this tool were added elements to assess the facilities access to WASH and surveillance materials. Data was collected by 6 trained surveyors with the help of a questionnaire administered to the head of each health facility and the support agent in charge of hygiene and sanitation. Data concerning items such as training, frequency or water interruption, frequency of toilet cleaning was collected by interview. Data on the availability of resources and materials for surveillance and case management, access to water, sanitation, and hygiene was recorded after direct observation by surveyors; see Questionnaire (Additional file 1).

### Data management

Data collected was analyzed with stata 11. Data was keyed and cleaned prior to analysis in excel. Statistical analysis done here were essentially descriptive in nature estimating the frequency of indicators like the proportion of health facilities with cholera reporting form, percentage of health facilities with ORS, percentage of health facilities with access to improved water sources, hygiene, and sanitation facilities. Data analysis was disaggregated by districts and regions to unveil any disparity existing among districts and between the two regions. Besides, factors associated with access to improved water sources were assessed using logistic regression in which the effect size was estimated with Odds Ratio(OR), with a 95% confidence interval (CI) and the *p*-value. The variables included in the final multiple logistic regression models were type of health facility, availability of budget for WaSH, Presence of Hygiene committee, and location on the health facility. These variables were simultaneously run in the model to estimate the effect size.

## Results

A total of 134 health facilities were assessed among which 26 (19.4%) in the Far North region (Kousseri and Mada health districts) and 108 (80.6%) in Littoral region (Deido and Nylon health districts). Private health facilities predominated in Douala contrarily to the Far North where public health facilities predominated. In all, most of the facilities (89/134) were health centers and integrated health centers. Also, half of the total facilities are opened 24 h per day every day in the Far North whereas more than 4/5 of the facilities are opened 24 h per day every day in Douala.

### Resources availability for case management and surveillance

Table 1 presents distribution of health facilities assessed stratified by district, region, type and category. One (0.7%) health facility in Mada Health district did not have a nurse. Table 2 summarizes the availability of resources and supplies for cholera surveillance and case management in various health facilities. One hundred (75%) health facilities had personnel trained on cholera surveillance in the last 12 months. Kousseri health district did not have many personnel trained on surveillance with 33.3% observed during the evaluation. Basic surveillance forms were not available in the majority of the health facility. Approximately 50% of the facilities in the Far North had cholera case definition pasted on the wall in the consultation room against 20% in the Littoral. Cholera case reporting form was available in approximately 40% of the health facilities.

**Table 1:** The distribution of health facilities assessed in terms of district, region, type, and category

Indicator	Modalities	Far North region						Littoral Region						Total	
		Kousseri (*N* = 15)		Mada (*N* = 11)		Total FN (*N* = 26)		Deido (*N* = 60)		Nylon (*N* = 48)		Total LT (*N* = 108)		LT + FN (*N* = 134)	
		N	%	n	%	N	%	n	%	n	%	N	%	n	%
Type of HF	Public	13	86.7	10	90.9	23	88.5	6	10.0	5	10.4	11	10.2	34	25.4
	Private	2	13.3	1	9.1	3	10.5	54	90.0	43	89.6	97	89.8	100	74.6
Category of HF	Health Centre	12	80.0	7	63.6	19	73	40	67.9	30	62.5	70	64.9	89	67.0
	Sub-divisional Hospital	1	6.7	3	27.3	4	15.4	5	8.5	6	12.5	11	10.2	15	11.3
	District Hospital	0	0.0	1	9.1	1	3.8	3	5.1	1	2.1	4	3.7	5	3.8
	Regional Hospital	1	6.7	0	0.0	1	3.8	1	1.7	0	0.0	1	0.9	2	1.5
	Central Hospital	0	0.0	0	0.0	0	0.0	2	3.4	0	0.0	2	1.9	2	1.1
	Others	1	6.7	0	0.0	1	3.8	8	13.6	11	22.9	19	17.6	20	15.0
Presence of a Medical doctor		2	13.3	1	9.1	3	11.5	29	48.3	28	58.3	57	52.8	60	44.8
Presence of a Nurse		15	100.0	10	90.9	25	96.1	60	100.0	48	100.0	108	100.0	133	99.3
Presence of a lab Technician		9	60.0	3	27.3	12	46.1	55	91.7	42	87.5	97	89.8	109	81.3
Presence of cholera specific bed		1	6.7	0	0.0	1	3.8	1	1.7	1	2.1	2	1.9	3	2.2
Presence of an isolation room		5	33.3	2	18.2	7	26.9	5	8.3	1	2.1	6	5.6	13	9.7
Presence of hospitalization room		12	80.0	8	72.7	20	76.9	59	98.3	47	97.9	106	98.1	125	94.0
Presence of shift services		8	55.3	6	54.3	14	53.8	55	91.7	41	85.4	96	88.9	110	82.1
HF opened 24/24H all year round		8	53.3	5	45.5	13	50.0	55	91.7	43	89.6	98	90.7	111	82.8

**Table 2:** The availability of resources and supplies for cholera surveillance and case management in the health facilities.

Indicator	Modalities	Far North region						Littoral Region						Total	
		Kousseri(N = 15)		Mada(N = 11)		Total FN(N = 26)		Deido(N = 60)		Nylon(N = 48)		Total LT(N = 108)		LT + FN(N = 134)	
		N	%	n	%	N	%	n	%	n	%	N	%	n	%
HF with personnel trained in the last 12 months	Surveillance	5	33.3	11	100.0	16	61.5	43	71.7	41	85.4	84	77.8	100	74.6
	Case management	7	46.7	11	100.0	18	69.1	2	3.3	6	12.5	8	7.4	26	19.4
Presence of supplies for case management	ORS	6	40.0	2	18.2	8	30.8	58	96.7	36	75.0	94	87.0	92	68.7
	Ringer lactate	9	60.0	8	72.7	17	65.4	48	80.0	48	100.0	96	88.9	123	91.8
	Zinc	1	6.7	0	0.0	1	3.8	17	28.3	22	45.8	39	36.1	40	29.9
	Syringe and perfuser	10	66.7	9	81.8	19	73.1	58	96.7	48	100.0	106	98.1	125	93.3
	Catheter for adult	9	60.0	9	81.8	18	69.2	48	80.0	46	95.8	94	87.0	122	91.0
	Catheter for children	10	66.7	9	81.8	19	73.1	47	78.3	46	95.8	93	86.1	122	91.0
	Doxycycline	7	46.7	7	63.6	14	53.8	48	80.0	42	87.5	90	83.3	104	77.6
	Guide for case management	2	13.3	9	81.8	11	42.3	4	6.7	8	16.7	12	11.1	26	19.4
Presence of supplies for cholera surveillance	Case definition guide	4	26.7	9	81.8	13	50.0	15	25.0	7	14.6	22	20.4	35	26.1
	Case reporting form	2	13.3	8	72.7	10	38.5	40	66.7	3	6.3	43	39.8	53	39.6
	Archived filled reporting form	2	13.3	10	90.9	12	46.2	37	61.7	1	2.1	38	35.2	50	37.3
	Investigation form	2	13.3	9	81.8	11	42.3	5	8.3	2	4.2	7	6.5	16	11.9
	Archived filled investigation form	2	13.3	7	63.6	9	34.6	1	1.7	1	2.1	2	1.9	13	9.7
	Rapid cholera diagnostic test	1	6.7	0	0.0	1	3.8	1	1.7	7	14.6	8	7.4	9	6.7
	CaryBlaire	0	0.0	0	0.0	0	0.0	1	1.7	1	2.1	2	1.9	2	1.5
	Stool bottle	8	53.3	8	72.7	16	61.5	51	85.0	36	75.0	87	80.6	103	76.9
	Swab sticks	2	13.3	4	36.4	6	23.1	54	90.0	40	83.3	94	87.0	100	74.6

From table 1, 26(19.4%) health facilities had personnel trained on cholera case management in the last 12 months with a great disparity between the Far North (69.1%) and the Littoral (7.4%). Lifesaving supplies were not available in all health facilities with 30% in the Far North against 87.0% in Littoral having ORS during the evaluation. However, 123 (91.8%) facilities had Ringer lactate solution which can equally be used for patient rehydration when in need. Most health facilities did not have isolation room, or even beds reserved for patients with cholera (90.3 and 97.8% respectively). Case management guideline was not available in 108(80.6%).

### Access to water

Table 3 presents the distribution of access to improved sources of water in the health facilities. A total of 7(5.1%) health facilities either relied on unimproved water sources or did not have any source at all. The results suggests that health facilities in Littoral had better access to improved water sources compared to those in the Far North region. On the other hand, 56.6% of the health facilities had a secondary source of drinking water different from the main source with 19.4% of the secondary sources being improved. The frequency and duration of water interruption was not negligible.

**Table 3:** Distribution of access to drinking water of the health facilities

Indicator	Modalities	Far North region(FN)						Littoral region(LT)						Total	
		Kousseri (N = 15)		Mada (N = 11)		Total FN (N = 26)		Deido (N = 60)		Nylon (N = 48)		Total LT (N = 108)		LT + FN (N = 134)	
		N	%	n	%	n	%	n	%	n	%	n	%	n	%
Main water source at HF	CDE(pipe borne water)	3	20.0	1	9.1	4	15.4	55	91.7	24	50.0	79	73.1	83	61.9
	Borehole	10	66.7	8	72.7	18	69.2	5	8.3	21	43.8	26	24.1	44	32.8
	Unprotected Well	1	6.7	1	9.1	2	7.7	0	0.0	2	4.2	2	1.9	4	3.0
	Lake/River	0	0.0	1	9.1	1	3.8	0	0.0	0	0.0	0	0.0	1	0.7
	Others	0	0.0	0	0.0	0	0.0	0	0.0	1	2.1	1	0.9	1	0.7
	Does not exist	1	6.7	0	0.0	1	3.8	0	0.0	0	0.0	0	0.0	1	0.7
Main water source at HF	Improved	13	86.7	9	81.8	22	84.6	60	100.0	45	93.8	105	97.2	127	94.8
	Unimproved	1	6.7	2	18.2	3	11.5	0	0.0	3	6.3	3	2.8	6	4.5
	Does not exist	1	6.7	0	0.0	1	3.8	0	0.0	0	0.0	0	0.0	1	0.7
Secondary water source	Present	7	46.7	6	54.5	13	50.0	18	30.0	27	56.3	45	41.7	76	56.7
	Improved	4	26.7	4	36.4	8	30.8	9	15.0	9	18.8	18	16.7	26	19.4
Frequency of main source interruption	< 1 day	12	80.0	9	81.8	21	80.8	48	80.0	33	68.8	81	75.0	102	76.1
	1–7 days	3	20.0	2	18.2	5	19.2	12	20.0	15	31.2	27	35.0	32	23.9
	> 7 days	0	0.0	0	0.0	0	0.0	0	0.0	0	0.0	0	0.0	0	0.0
Mean duration of Main source interruption	< 1 day	13	86.7	9	81.8	22	84.6	51	85.0	34	70.8	85	78.7	107	79.9
	1–7 days	2	13.3	2	18.2	4	15.4	9	15.0	14	29.2	23	21.3	27	20.1
	> 7 days	0	0.0	0	0.0	0	0.0	0	0.0	0	0.0	0	0.0	0	0.0

Table 4 shows the determinants of access to improved water sources. Urban (OR = 6.4, *p*-value = 0.021) and private (OR = 8.4, p-value = 0.013) health facilities were significantly more likely to have an improved water source from univariate analysis but when run in a multivariate logistic regression, no statistical significance was observed in any of the factors. It is worth noting that all the 7 health facilities that did not have improved water sources were all health centers and integrated health centers.

**Table 4:** Determinants of access to improved water source

	Univariate analysis				Multivariate analysis			
Factor	OR	95% CI		p-value	OR	9CI%		p-value
Health facility location? (Urban/Rural)	6.36	1.329361	30.46266	0.021*	2.07	0. .268855	15.989020	0.48
Presence of budget for WaSH? (Yes/No)	2.09	0.391669	11.21225	0.387	1.04	0.131814	8.234411	0.97
Presence of hygiene committee?(Yes/No)	3.61	.4220979	30.96676	0.241	2.91	0.220491	38.452590	0.42
Type of health facility (Private/public)	8.45	1.55676	45.84738	0.013*	5.04	0. .586502	43.355700	0.14
Health center or integrated health centers(Yes/No)	**	**	**	**	*Was not included in the model*			

*statistically significant; **could not be computed because a cell had zero (0) as the value. *The variables included in the final model were type of health facility, availability of budget for WaSH, Presence of Hygiene committee, and location on the health facility*

### Hygiene

Table 5 summarizes the situation of hygiene in the health facilities. A total of 48(36.4%) health facilities had a committee in place in charge of sanitation and hygiene. There was a large disparity between the Far North and Littoral with 11% having hygiene committee in the Far North against 42% in the Littoral. More than 95.4% health facilities did not have the protocol for hand washing and 45% did not have soap for hand washing. A washbasin was present in 54.5% of the facilities and 44.7% had a budget allocated for hygiene and sanitation of the facilities. The absence of budget allocated for hygiene and sanitation was highest in the Koussseri health district with 1 (6.7%) having such budget. Less than one-tenth (1/10) of the health facilities had hand washing protocol pasted on the wall during the assessment. Close to 90% had chlorine bleach for disinfection and 55% declared to wash the toilet two and more times per week.

**Table 5:** The situation of hygiene of health facilities

Indicator	Far North Region (FN)						Littoral region (LT)						Total	
	Kousser i(N = 15)		Mada (N = 11)		Total FN (N = 26)		Deido (N = 60)		Nylon (N = 48)		Total LT (N = 108)		LT + FN(N = 134)	
	N	%	n	%	N	%	n	%	N	%	n	%	n	%
Presence of a hygiene committee	2	13.3	1	9.1	3	11.5	13	22.4	32	66.7	45	42.5	48	36.4
Presence of a budget allocated to Hygiene/sanitation	1	6.7	4	36.4	5	19.2	22	37.9	32	66.7	54	50.9	59	44.7
Presence of a support agent(cleaner)	6	40.0	6	54.5	12	46.2	33	55.9	29	60.4	62	57.9	74	55.6
Presence of washbasin	12	80.0	4	36.4	16	61.5	38	63.3	19	39.6	57	52.8	73	54.5
Presence of soap for hand washing	10	66.7	11	100.0	21	80.8	41	68.3	12	25.0	53	49.1	74	55.2
Presence of a guideline on hand washing	6	40.0	5	45.5	9	34.6	18	30.0	4	8.3	12	11.1	21	24.6
Presence of a cleaning time table	13	86.7	4	36.4	17	65.4	30	51.7	40	85.1	70	66.7	87	66.4
Toilet washed two or more times per day	6	42.9	3	27.3	9	36.0	35	59.3	28	60.9	60	60.0	72	55.4
Presence of chlorine bleach for disinfection	7	46.7	6	66.7	13	54.2	57	98.3	46	95.8	103	97.2	116	89.2
Water treated before using for hand washing	11	78.6	9	90.0	20	83.3	12	20.0	28	58.3	40	37.0	60	45.5
Water treated before using for cleaning tools	12	80.0	9	90.0	21	84.0	20	33.3	31	64.6	51	47.2	72	54.1

### Sanitation

Table 6 presents the availability of sanitary facilities in the health facilities. A total of 4(3.0%) health facilities did not have a toilet and all of them were in the Far North region. Also, 109 (82.0%) health facilities had a good system of evacuating used water whereas on 38(28.4%) had a septic tank. Septic tank was present in many health facilities of the Far North (80.8%) than those of the Littoral (15.7%). Furthermore, guidelines on waste management were present in less than 20% of the facilities and 31.3% had a personnel trained on waste management. Less than one-tenth of the health facilities in Mada had a person trained on waste management.

**Table 6:** Availability of sanitary facilities in the health facilities

Indicator	Far North region(FN)						Littoral region(LT)						Total	
	Kousser i(N = 15)		Mada (N = 11)		Total FN (N = 26)		Deido (N = 60)		Nylon (N = 48)		Total LT(N = 108)		LT + FN (N = 134)	
	n	%	N	%	n	%	n	%	N	%	n	%	N	%
Does not have any toilet	2	13.3	2	18.2	4	15.4	0	0.0	0	0.0	0	0.0	4	3.0
Presence of an adequate system for evacuation of used water	7	46.7	2	20.0	9	36.0	57	95.0	43	89.6	100	92.6	109	82.0
Presence of an incinerator	2	13.3	1	12.5	3	13.0	2	3.3	3	6.3	5	4.6	8	6.1
Presence of autoclave	1	6.7	2	18.2	3	11.5	19	31.7	13	27.1	32	29.6	35	26.1
Presence of sterilizer	3	20.0	3	27.3	6	23.1	33	55.0	26	54.2	59	54.6	65	48.5
Presence of septic tank	12	80.0	9	81.8	21	80.8	12	20.0	5	10.4	17	15.7	38	28.4
Presence of a guideline on the treatment of biological waste	1	6.7	2	20.0	3	12.0	18	30.0	4	8.3	22	20.4	25	18.8
Presence of a personnel trained on waste treatment	4	26.7	1	9.1	5	19.2	14	23.3	23	47.9	37	34.3	42	31.3
waste separated (ie, sharps, infectious waste, and non-infectious waste)	0	0.0	0	0.0	0	0.0	26	43.3	29	60.4	55	50.9	55	41.0

## Discussion

This article describes the readiness of health facilities to respond to a sudden cholera outbreak in cholera-prone districts in Cameroon. The results reveal a number of weaknesses in the health facilities regarding cholera surveillance, case management, and access to WaSH. These weaknesses can greatly hinder the capacity of these health facilities to respond correctly and promptly to an outbreak of cholera.

For an effective cholera outbreak response, the surveillance system in place must first of all detect the outbreak on time [15–17]. Though close to 75% of the health facilities had personnel trained on cholera surveillance in the last 12 months, most of them were working in urban health facilities in Douala. Interestingly, only 33% facilities had trained personnel in Kousseri Health district which is one of the most affected districts during recent epidemics [2, 19]. Also, some essential surveillance materials like case definition, reporting forms, investigation forms, and stool bottles were still lacking in the majority of the facilities. These results are in agreement with the results of previous studies which unveiled challenges and weaknesses of surveillance in Cameroon and Bangladesh [20–22]. The consequences of this can be a delay in the detection and hence response. Any delay in the detection of a cholera outbreak can delay the outbreak response and complicate the outbreak due to the contamination of many water sources hence multiplication of sources of transmission, and prolonging the response duration [23–25]. All health facilities in these districts must be supplied with basic materials and trained on surveillance to ensure that outbreaks are detected on time.

Case management is very important in ensuring the reduction of case fatality during cholera outbreaks. The results show that many health facilities lacked basic lifesaving supplies like the ORS and case management guide. Also less than 20% of these facilities had personnel trained on cholera case management in the last 12 months. This is a very serious situation especially in the Far North region where more than 70% of the health facilities did not have ORS since this region is always affected by most (if not all) cholera outbreaks and other enteric diseases in Cameroon [4, 6, 21]. The lack of ORS can lead to alarming death rate during an outbreak [26–28]. According to the WHO, the correct use of ORS alone can maintain the case-fatality during an outbreak to less than 1% [23, 28]. This can be a possible explanation to the high deaths rate observed in these districts during previous cholera outbreaks [24]. However, most facilities (91%) had ringer lactate solution which is used in the management of moderate to severe cases of cholera and this can be critically useful in reducing the case fatality rate during outbreak. All health facilities in the four districts should be provided with essential supplies especially ORS and management guide to ensure proper management of cases in case of any outbreak so as to minimize case fatality.

Access to potable water is essential to ensure good hygiene and sanitation and to limit nosocomial infection. This is underlined in the United Nations’ Sustainable Development Goal 6(SDG6). Based on the results, access to water in these health facilities still required a lot of improvements. Though more than 95% of the health facilities were supplied with improved sources of drinking water, 7(5.1%) of the health facilities either relied on unimproved sources as their main sources of drinking water or did not have any source at all. Also, only 19.4% had improved secondary sources different from the main source. Urban and private health facilities were significantly more likely to have an improved water source. Besides, all health facilities reported frequent interruption of their water sources with some having and average duration of about 3 days/per week with no water. Low access to water at health facilities has be documented in other studies [12, 25]. During cholera outbreaks, all patients are polled to the health facilities and those facilities without improved sources of water can easily turn to ‘transmission milieus’ due to lack of water to ensure proper hygiene and sanitation [29, 30]. This can be very serious especially because patients are generally considered weak and vulnerable to infection [17]. This can increase the attack and death rates among in-patients, out-patients and even among health staffs to an extent. All health facilities should be provided with at least one source of water to ensure that the hygiene and sanitation conditions are up to standard so as to reduce the rate of infection and improve patient’s management and hence outcomes.

Access to water, sanitation and hygiene is a key component of the Sustainable Development Goal (SDG6) and this should be achieved both at community and health facilities levels so as to ensure an extended impact and sustainability. The results reveal a serious problem regarding hygiene and sanitation in these health facilities. In fact, more than 95% health facilities did not have the protocol for hand washing. Approximately 3% did not even have a toilet. To further worsen the situation, close to 56% did not have any budget allocated for hygiene of the facilities and 45% did not have soap for hand washing. Poor hygiene and sanitation in hospitals have been documents in a number of studies [31, 32]. Hand washing with soap is a very important component of body hygiene. Simple hand washing with soap has been proven to be effective in reducing infections [33–36]. The protocol for hand washing and soap are very essential to ensure that staffs wash their hands correctly before medical interventions and can therefore contribute to reduce nosocomial infections. In the same line, the lack of toilet, septic tank, etc. coupled with no budget allocated for hygiene and sanitation can lead to compromised hygiene and waste management which turn to favor the transmission of diseases in the hospital milieu [37–39].

This study did not collect data on all aspect of health facility preparedness like rate of supervision and community sensitization and the causes of the weaknesses observed were not investigated. Concerning supplies, we did not collect data on the quantity present which could be useful in estimating the capacities of health facilities that had these supplies in management the first cases of cholera during outbreak. Also, we did not collect data on the type of toilet, water quality, or investigate if facilities without a water source has source situated within 0.5KM from it. Furthermore, statistical test power was low due to low sample size. However, the results are largely informative and usable to identify priority interventions to improve cholera control in the study area.

## Conclusion

The health facilities in these four districts have weaknesses in their capacity to cope with a cholera epidemic. These are grouped under the capacity in surveillance of cholera and case management and access to WaSH. In terms of surveillance, these facilities had few trained personnel and lacked basic surveillance materials and tools. This was most prominent in rural districts where cholera outbreak is most frequent. The ability of health facilities in case management need improvement in the training of personnel and prepositioning of supplies like ORS. To make things worse, poor access to WaSH was equally uncovered in many health facilities. Some facilities did not have any water source or relied on surface water like lake/river and others lacked toilets. Theses weaknesses can compromise the ability of the health facility to respond to cholera outbreak and can cause high case fatality of cholera during outbreaks. To improve on the readiness of these districts on cholera outbreak response, the following recommendations are made:

### To the heads of health facilities, district health services and regional delegation of public health

- Train and regularly supervise health facilities personnel on cholera surveillance and Train and regularly supervise health facilities personnel on cholera surveillance and equally ensuring that all health facilities are provided with surveillance tools. This should include case definition, investigation form, stool bottle.
- Train and regularly supervise health facilities personnel on cholera case management and equally ensuring that all health facilities are provided with necessary supplies in sufficient quantities. This should include ORS, ringer lactate, management protocol,
- Ensure that essential supplies for hygiene such as soap, chlorine bleach for disinfection, protocol for hand washing, and washbasin are always in the health facilities at all times.

### To the Ministry of Public Health

- To ensure all health facilities are provided with at least one improved source of drinking water, and a toilet.
- The level of preparedness of health facilities in other districts at risk of cholera should be evaluated in terms of the availability and training of human resource, equipment, supplies, access to WaSH, and guidelines and ensure that the identified weaknesses are corrected.

## Additional file

Additional file 1: Questionnaire, this is the data collection tool that was used in the data collection for this study. (DOCX 26 kb)

## Data Availability

The datasets used and/or analyzed during the current study are available from the corresponding author on reasonable request.

## Abbreviations

CI: Confidence interval
CMA: Subdivisional Hospital
HF: Health facility
IHC: Integrated health center
MPA: Minimum Package of Activities
OR: Odds ratio
SDG: Sustainable development Goal
WaSH: Water sanitation and hygiene
