# Supplementary material for: Health facility preparedness for cholera outbreak response in four cholera-prone districts in Cameroon: a cross sectional study
Source: BMC Health Serv Res. 2019 Jul 8;19:458. doi: 10.1186/s12913-019-4315-7 (PMC6615310; doi:10.1186/s12913-019-4315-7)
Supplement: Supplementary file 1 — Questionnaire, this is the data collection tool that was used in the data collection for this study. (DOCX 26 kb) [file 12913_2019_4315_MOESM1_ESM.docx]

***Reducing the morbidity and mortality of cholera in Cameroon (REMMOCC)***

**Preparedness of health facility for cholera outbreak response**

| No | Question | Comments |
| --- | --- | --- |
|  | Date |  |
|  | Identification and characteristics of the health facility |  |
|  | District  *Kousseri =1, Mada =2, Deido =3, Nylon=4* |  |
|  | Health area |  |
|  | Healthfacility |  |
|  | Type of HF  Public =1 ; private =2 ; confessional =3 |  |
|  | Category of HF  HC =1 ; IHC =2 ; CMA =3 ; DH =4 ; RH =5 ; central hospital =6 ; reference hospital =7 ; others =8 |  |
|  | Position of Person interview |  |
|  | Contact of Person interview |  |
|  | Is there a ‘service de garde/ astreinte’ |  |
|  | Is the facility opened 24/24H and 365/365 days |  |
|  | *Number of personnel (including support agents)* |  |
|  | *Number trained on case management* |  |
|  | *Number trained on epidemiological surveillance* |  |
|  | *Number of MD* |  |
|  | *Number of nurses and assistance nurses* |  |
|  | *Number of laboratory technicians* |  |
|  | *Number of consultation per day* |  |
|  | *Number of patient hospitalized per day* |  |
|  | *Number of patient career per day* |  |
|  | *Apart from patient and their careers, how many people from the community fetch water here at the centre* |  |
|  | *Number of bed* |  |
|  | *Number of beds specific for cholera patients* |  |
|  | *Number of hospitalization rooms* |  |
|  | *Number of services* |  |
|  | *Is there a room for isolation of cholera cases*  *Yes =1, no =2* |  |
|  | *Number of bed in the isolation room* |  |
|  | *Number of deliveries per day* |  |
|  | *Number of surgical operations per day* |  |
|  | *Presence of a hygiene committee?*  *Yes =1, no =2* |  |
|  | *Presence of cleaner*  *Yes =1, no =2* |  |
|  | *Does the health facility have a budget line for WASH?*  *Yes =1, no =2* |  |
|  | *Do you face difficulties obtaining the necessaries for WASH?(ask to the cleaner)*  *Yes =1, no =2* |  |
|  | Hygiene |  |
|  | Is there a guideline of hands hygiene?  *Yes =1, no =2* |  |
|  | Is there a program for cleaning the hospital?  *Yes =1, no =2* |  |
|  | At what frequency is the toilet cleaned?  *<2x/day =1, 2x/day (and when it is dirt) =2* |  |
|  | Water |  |
|  | Principal source of water  CDE =1, forage =2, well =3, spring =4, river =5, lake =6, other =7, does not exist =8 |  |
|  | Is the principal source improved? (observe)  *Yes =1, no =2* |  |
|  | Is there any secondary source?  *Yes* CDE =1; yes forage =2; yes well =3; yes spring =4;yes river =5; yes lake =6;yes other =7*; no =8* |  |
|  | Is the secondary source improved?  *Yes =1, no =2* |  |
|  | Number of water sources for the Facility |  |
|  | Number of functional water sources for the Facility |  |
|  | What is the frequency of interruption of the main source of water in days (if less than one day, write zero ‘0’) |  |
|  | Normal duration of interruption in days (if less than one day, write zero ‘0’) |  |
|  | The longest duration of interruption ever experienced in days (if less than one day, write zero ‘0’) |  |
|  | Do you have containers (reservoirs) to conserve potable water at centre?  *Yes =1, no =2* |  |
|  | Total volume of all these containers joint in litres( if no container, write zero ‘0’) |  |
|  | Are all containers covered with their covers with taps?  *Yes =1, no =2* |  |
|  | How long can you use water stored in these containers if there interruption? (in days) |  |
|  | Do you judge sufficient the quantity of water available for your activities?  *Yes =1, no =2* |  |
|  | Do you treat drinking water?  *Yes =1, no =2* |  |
|  | Sanitation |  |
|  | Number of functional toilet? |  |
|  | Type of functional toilet  Flushing =1, pit toilet =2, others =3 |  |
|  | Do you store water in the toilet?  *Yes =1, no =2* |  |
|  | Is there adequate evacuation system for used water?( should be done by an impermeable tube to impermeable septic fosse)  *Yes =1, no =2* |  |
|  | Presence of incinerator  *Yes =1, no =2* |  |
|  | How do you eliminate wastes with sharp objects? |  |
|  | How do you eliminate waste that are infectious which are not sharp object? |  |
|  | How do you eliminate general waste |  |
|  | Presence Case definition of a suspected cholera pasted on the wall? |  |
|  | Cholera management guideline pasted |  |
|  | Presence of form for reported cases |  |
|  | Presence of reporting forms |  |
|  | Presence of investigation form |  |
|  | Presence of form for investigated forms |  |

| Do you have | Encircle the right answer | | Quantity available | Quantity order |
| --- | --- | --- | --- | --- |
| Ringer lactate solution? | Yes | No |  |  |
| ORS? | Yes | No |  |  |
| Zinc? | Yes | No |  |  |
| Intravenous tubes and syringe? | Yes | No |  |  |
| Catheter for adult? | Yes | No |  |  |
| Catheter for children | Yes | No |  |  |
| Dipstick | Yes | No |  |  |
| Doxycycline? | Yes | No |  |  |
| Chlorine water? | Yes | No |  |  |
| Caryblaire? | Yes | No |  |  |
| Tools bottle | Yes | No |  |  |
| (Slaps)Ecouvillons | Yes | No |  |  |

Observation grid

|  | Toilets | | | | | Bathrooms | | Hand washing | | | | Wastes | | Beds | |
| --- | --- | --- | --- | --- | --- | --- | --- | --- | --- | --- | --- | --- | --- | --- | --- |
| Room/hall | Total (#) | Clean (#) | Functional (#) | Trashcan (#) | Hand washing (#) | Total (#) | Functional (#) | Total (#) | Functional (#) | Soap (#) | poster (#) | 3 separate trashcans (yes/no) | Well stored (yes/no) | Total (#) | Mosquito net (#) |
|  |  |  |  |  |  |  |  |  |  |  |  |  |  |  |  |
|  |  |  |  |  |  |  |  |  |  |  |  |  |  |  |  |
|  |  |  |  |  |  |  |  |  |  |  |  |  |  |  |  |
|  |  |  |  |  |  |  |  |  |  |  |  |  |  |  |  |
|  |  |  |  |  |  |  |  |  |  |  |  |  |  |  |  |
|  |  |  |  |  |  |  |  |  |  |  |  |  |  |  |  |
|  |  |  |  |  |  |  |  |  |  |  |  |  |  |  |  |
|  |  |  |  |  |  |  |  |  |  |  |  |  |  |  |  |
|  |  |  |  |  |  |  |  |  |  |  |  |  |  |  |  |
|  |  |  |  |  |  |  |  |  |  |  |  |  |  |  |  |
|  |  |  |  |  |  |  |  |  |  |  |  |  |  |  |  |
|  |  |  |  |  |  |  |  |  |  |  |  |  |  |  |  |
|  |  |  |  |  |  |  |  |  |  |  |  |  |  |  |  |
|  |  |  |  |  |  |  |  |  |  |  |  |  |  |  |  |
|  |  |  |  |  |  |  |  |  |  |  |  |  |  |  |  |
|  |  |  |  |  |  |  |  |  |  |  |  |  |  |  |  |
|  |  |  |  |  |  |  |  |  |  |  |  |  |  |  |  |
|  |  |  |  |  |  |  |  |  |  |  |  |  |  |  |  |
|  |  |  |  |  |  |  |  |  |  |  |  |  |  |  |  |
